# *Lactobacillus acidophilus* UCLM‐104 and *Lacticaseibacillus paracasei* UCLM‐41 Are Promising Candidates to Produce Synbiotic Yogurt

**DOI:** 10.1002/fsn3.70539

**Published:** 2025-06-30

**Authors:** Sara Rodríguez‐Sánchez, Pilar Fernández‐Pacheco, Cristina de los Reyes‐Ramos, Emma Burgos‐Ramos, Susana Seseña, M. Llanos Palop

**Affiliations:** ^1^ Departamento de Química Analítica y Tecnología de Alimentos, Facultad de Ciencias Ambientales y Bioquímica Universidad de Castilla‐La Mancha (UCLM) Toledo España; ^2^ Departamento de Química Inorgánica, Orgánica y Bioquímica, Facultad de Ciencias Ambientales y Bioquímica Universidad de Castilla‐La Mancha (UCLM) Toledo España

**Keywords:** dairy products, functional foods, lactic acid bacteria, probiotic, synbiotic

## Abstract

Functional foods consumption, included the synbiotics, has garnered attention of the consumers because of their health's benefits, being lactic acid bacteria (LAB) the microorganisms most used for their manufacturing. Twelve potential probiotic LAB strains were evaluated for cholesterol removal, phenolic content, antioxidant properties, α‐glucosidase inhibitory activity (anti‐diabetic effect), and in vitro angiotensin‐I‐converting enzyme‐ (ACE) inhibitory activity (anti‐hypertensive effect), together with the assimilatory capacity of different prebiotics such as inulin, lactulose, β‐glucans, and fructooligosaccharides, to be used in the production of a synbiotic yogur. Their anti‐inflammatory potential in Caco‐2 cells was also assayed. *Lactobacillus* (*Lact*.) *acidophilus* UCLM‐104 and *Lacticaseibacillus* (*Lc*.) *paracasei* UCLM‐41, exhibited interesting health promoting properties and an effective modulation of inflammation. Sensory analysis of the synbiotic yogurts manufactured with each of the strains displayed no significant differences between them, but there were statistically significant differences with the control yogurt, garnering greater acceptance from taste‐testers. Therefore, both strains emerge as promising probiotic candidates to be used to produce synbiotic yogurts with potential health‐promoting attributes and sensory appeal. This study represents a pioneering endeavor, being the first to comprehensively evaluate the health‐enhancing properties of potential probiotic strains and subsequently apply them in the production of synbiotic yogurts.

## Graphical abstract

Use of health‐promoting lactic acid bacteria in the production of synbiotic yogurt. *Lactobacillus acidophilus* UCLM‐104 and *Lacticaseibacillus paracasei* UCLM‐41 are promising probiotic strains due to their antioxidant, cholesterol removal, phenolic content, ACE‐inhibition, α‐glucosidase inhibitory activity, and anti‐inflammatory potential.

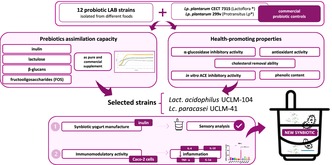

## 1 Introduction

Changes in lifestyle, including improper nutrition, physical inactivity, and other unhealthy habits, are promoting an increase in the prevalence of non‐infectious chronic diseases such as cardiovascular diseases, diabetes, and obesity, among others, which are the leading causes of death worldwide (World Health Organization (WHO) 2022). To slow down and reverse this fatal situation, international health organizations, such as the Spanish Society of Community Nutrition, recommend dietary changes, including the consumption of functional foods (Aranceta‐Bartrina et al. 2019).

Functional foods are defined as natural or processed foods containing biologically active compounds that provide documented health benefits and/or can reduce the risk of suffering from non‐infectious diseases, providing benefits in addition to the foods' nutritional properties (Duttaroy 2019). In the realm of functional foods, probiotics have established a dominant presence, constituting a significant portion of the overall functional foods market; however, it is essential to highlight the potential of other functional foods such as synbiotics, which are defined as a mixture comprising live microorganisms and substrate(s) selectively utilized by host microorganisms, that confer a health benefit for the host (Swanson et al. 2020).

The main microorganisms used as probiotics in the manufacturing of functional foods are lactic acid bacteria (LAB) (Florou‐Paneri et al. 2013), with the species *Lactobacillus* (*Lact*.) *acidophilus*, *Lacticaseibacillus* (*Lc*.) *paracasei*, and *Lactiplantibacillus* (*Lp*.) *plantarum* being the most commonly used due to their interesting health‐promoting properties (Rodríguez‐Sánchez, Fernández‐Pacheco, et al. 2021). Most of the clinical evidence substantiating the health benefits of probiotics are through viable cells (Jäger et al. 2018) and the metabolites produced during fermentation (Somashekaraiah et al. 2019), although there is increasing evidence that the non‐viable probiotic cells also confer health benefits (Cuevas‐González et al. 2020). LAB are also a potential source of antioxidants, such as phenolic compounds (Rizzello et al. 2017), which can protect cells against oxidative stress by reactive oxygen species (ROS), inhibiting the development of diseases such as cancer, atherosclerosis, and inflammatory diseases (Hill et al. 2018).

Other important properties of probiotics include cholesterol‐removal ability, which occurs by different mechanisms (Kumar et al. 2012), preventing the development of cardiovascular diseases (Remagni et al. 2013), as well as anti‐diabetic and anti‐hypertensive effects. The anti‐diabetic effect results from the inhibition of α‐glucosidase activity, which modifies the intestinal absorption of carbohydrates, lowering blood glucose levels (Chapman et al. 2011), and the anti‐hypertensive effect is a consequence of the release of bioactive peptides from the casein proteolysis of milk, which can inhibit the angiotensin‐I‐converting enzyme (ACE) (Tagliazucchi et al. 2020). This enzyme carries out the transformation of the hormone, angiotensin I, into the potent vasoconstrictor, angiotensin II, playing a key role in the control of blood pressure (Fuglsang et al. 2002; Rubak et al. 2020). Finally, there is growing evidence that probiotic bacteria can interact with cytokines and stimulate their production in intestinal cells, contributing to immunological homeostasis (Zheng et al. 2020). Therefore, the use of probiotics with immunomodulatory properties represents a promising approach for enhancing immunological responses to diseases, including infectious and inflammatory ones. In this respect, it has been reported that probiotic administration can down‐regulate intestinal inflammation in patients suffering from inflammatory bowel diseases (Mazziotta et al. 2023).

The objective of this research was to evaluate 12 LAB strains for their health‐promoting properties, with a specific focus on their suitability for incorporation into the production of novel synbiotic yogurts possessing desirable sensory characteristics.

## 2 Materials and Methods

### 2.1 Bacterial Strains and Growth Conditions

Twelve LAB strains from the collection of the Microbiology Laboratory of the University of Castilla‐La Mancha (Toledo, Spain) were analyzed. They were genotyped and identified as belonging to *Lactiplantibacillus* (*Lp*.) *plantarum* (UCLM‐36, UCLM‐37, UCLM‐72, UCLM‐76, UCLM‐93, and UCLM‐107), *Levilactobacillus* (*Lv*.) *brevis* (UCLM‐99 and UCLM‐111), *Lactobacillus* (*Lact*.) *acidophilus* (UCLM‐104), *Lact*. *delbrueckii* (UCLM‐32), and *Lacticaseibacillus* (*Lc*.) *paracasei* (UCLM‐24 and UCLM‐41) species (Nieto‐Arribas et al. 2009; Pérez‐Martín et al. 2014) and were selected based on their safety and survival to simulated gastrointestinal (GI) tract conditions (Rodríguez‐Sánchez, Fernández‐Pacheco, et al. 2021; Rodríguez‐Sánchez, Ramos, et al. 2021).

*Lactococcus lactis* subsp. *lactis* A0W5, a highly proteolytic strain from our own collection, was used as a control in the in vitro ACE‐inhibition assay, and the commercial probiotic strains, *Lp. plantarum* CECT 7315 from Lactoflora Protector Inmunitario (Laboratorio Stada, Barcelona, Spain) and *Lp. plantarum* 299v from Protransitus Lp (Laboratorios Salvat, Barcelona, Spain), were used for comparison in health‐promoting properties assays.

Pure cultures of the strains in MRS broth (Pronadisa, Madrid, Spain) were maintained frozen at −80°C, supplemented with 20% (v/v) glycerol as a cryoprotectant, and before being assayed, were revitalized by inoculation (10%) in MRS broth, and then underwent aerobic incubation at 30°C for 24 h.

### 2.2 Prebiotic‐Assimilation Capacity

LAB strains were grown by using a modified MRS broth (m‐MRS) without glucose, but with 1% (w/v) of a prebiotic. The prebiotic compounds assayed were inulin, lactulose, β‐glucan, and fructooligosaccharides (FOS), and they were acquired in two forms: as pure compounds (p) from Sigma‐Aldrich (St. Louis, MO, USA) and as a commercial supplement(s) without added sugars from SaludViva (Alicante, Spain), Lainco (Barcelona, Spain), Energy Feelings (Tarragona, Spain) and Lamberts (Madrid, Spain), respectively.

Overnight cultures of each strain in MRS broth were centrifuged (18,500× *g* at 4°C for 15 min), and cells were washed twice in sterile phosphate buffer saline (PBS) at pH 7.2. They were then resuspended in PBS and used to inoculate 1% (v/v) m‐MRS broth in a P96 microtiter plate. The plate was incubated at 30°C for 48 h, and the optical density (OD) at 600 nm was measured every 30 min, with a previous agitation period of 15 s using the reader Synergy HT (Biotek, Vermont, USA). Growth curves were obtained by plotting OD_600_ versus time. The following parameters were calculated: lag phase (λ), generation time (G), specific growth rate constant (μ_max_), and the difference of OD_max_–OD_ini_. Each strain was assayed in triplicate.

### 2.3 Health‐Promoting Properties

Depending on the property assayed, overnight cultures of the LAB strains or their cell‐free supernatants (CFS) were used. To obtain CFS, the procedure described by Hamad et al. (2020) was followed. All the assays described below were performed in triplicate, except that of the immunomodulatory activity, which was in quadruplicate.

#### 2.3.1 Antioxidant Activity and Total Phenolic Content

The antioxidant activity was determined using the KMnO_4_‐agar method, as described by Hanchi et al. (2022) and the values were calculated by the subtraction of the diameters of the transparent halo for the assayed strain and that of the negative control (MRS broth).

The total phenolic content in the CFS was determined by the Folin–Ciocalteu spectrophotometric method, following the procedure described by Hamad et al. (2020). Results were expressed in terms of mg of gallic acid equivalent per L of supernatant, using the linear regression equation obtained from the standard gallic acid calibration curve (0–1500 mg/L).

#### 2.3.2 α‐Glucosidase Inhibitory Activity

The α‐glucosidase inhibitory activity of both intact cells and CFS was determined as reported by Ramos et al. (2022). The OD_405_ corresponding to the 4‐nitrophenol released was measured using the reader Synergy HT (Biotek). For each of these assays, a blank and a control were prepared, also as reported by these authors, using MRS broth for the control of the CFS assay.

#### 2.3.3 Cholesterol‐Removal Ability

MRS broth supplemented with 120 μg/mL of water‐soluble cholesterol and 0.3% (w/v) ox‐bile, both from Sigma‐Aldrich, was inoculated (1% v/v) with an overnight culture of the assayed LAB strain (8‐log colony forming units [cfu]/mL) and incubated at 30°C for 24 h.

The quantification of cholesterol was carried by the o‐phthalaldehyde (OPA) spectrophotometric method, as described by Ragul et al. (2017) using the linear regression equation obtained from the water‐soluble cholesterol calibration curve (0–120 μg/mL).

#### 2.3.4 Determination of in vitro ACE‐Inhibition and Peptide Content

Cells from an overnight culture of the LAB strain were harvested by centrifugation (18,500× *g* at 4°C for 15 min), washed with sterile PBS at pH 7.2, and re‐suspended in PBS to reach a turbidity adjusted to the point 3 of the McFarland standard (9‐log cfu/mL). A volume of 5 mL of 9.5% skimmed milk (w/v) was inoculated in triplicate with 50 μL of the prepared suspension to reach a concentration of around 7‐log cfu/mL and incubated at 30°C for 15 h. The measurement of the capacity of the LAB strains to inhibit the angiotensin‐I‐converting enzyme (ACE) in vitro was carried out in the supernatant of these milk cultures following the procedure described by Fuglsang et al. (2002).

For each of the samples, a blank was prepared, in which 20 μL of the ACE solution was replaced by water. In addition, both a control and a blank of the control were prepared. The first contained 280 μL of the N‐hippuryl‐His‐Leu hydrate (HHL, Sigma‐Aldrich) buffer and 20 μL of the ACE solution, and the second contained 280 μL of the HHL buffer and 20 μL of water.

The peptide content in the supernatant was quantified according to the OPA method, described by Church et al. (1983). The OD_340_ was measured, and the peptide content was expressed in terms of mg glycine per mL of supernatant, using the linear regression equation obtained from the glycine (Scharlab, Barcelona, Spain) calibration curve (0–5 mg/mL).

#### 2.3.5 Immunomodulatory Activity Assay

An assay to determine the anti‐inflammatory effect of the selected strains, as well as that of the two commercial probiotics strains, on LPS‐induced inflammation in Caco‐2 cells was carried out by measurement of mRNA levels of the pro‐inflammatory (TNF‐α and IL‐1α) and anti‐inflammatory (IL‐10 and IL‐4) cytokines.

The human adenocarcinoma Caco‐2 cell line was obtained from the American Type Culture Collection (Manassas, USA) and was routinely cultured in Dulbecco's modified Eagle's medium (DMEM), supplemented with 10% (v/v) foetal bovine serum (FBS) and 1% L‐Glutamine and penicillin–streptomycin, at 37°C in an atmosphere containing 5% CO_2_. Once Caco‐2 cells in the subculture passage between 33 and 42 reached 60% confluence (differentiated cells) (Natoli et al. 2012), they were seeded at a density of 2.5 × 10^5^ cells/well in a 48‐well tissue culture plate. The culture medium was changed every 2 days, and cells were grown for 7 days (Hidalgo et al. 1989).

Caco‐2 cells were starved in a medium without FBS or antibiotics before each treatment for 24 h. Half of the Caco‐2 cell culture was treated with LPS (10 μg/mL) from *Salmonella enterica* serovar *enteritidis* (MERCK‐ Millipore) for 24 h to induce inflammation in the cells (Wu, Huang, et al. 2019). Overnight cultures of the LAB and the commercial probiotics strains in MRS broth (8‐log cfu/mL) were centrifuged (18,500× *g* at 4°C for 10 min), and the pellet was resuspended in 0.3 mL of DMEM without FBS or antibiotics and added to the monolayer culture of Caco‐2 cells. The plates were incubated for 3 h, as previously described. After incubation, cells were washed with PBS, recollected, and stored at −80°C until use. The different combinations gave rise to 10 experimental groups. Each experiment was carried out in quadruplicate.

Total RNA from cells was obtained using the Qiazol lysis reagent (Qiagen, Hilden, Germany) by following the manufacturer's instructions. RNA concentration and purity were quantified using the NanoDrop 2000 spectrophotometer (Take3, BioTek, Vermont, USA) by calculating the ratio of the optical densities obtained at the wavelengths of 260/280 and 260/230 nm, respectively. Complementary DNA (cDNA) was synthesized from 1 μg of DNase‐treated RNA. Real‐time PCR was performed in duplicate in a total volume of 20 μL containing 5 μL of the RT reaction in an ABI Prism 7500 Fast Sequence Detection System (Applied Biosystem, Foster City, CA), as described by Visioli et al. (2022). The real‐time PCR was performed under thermal‐cycling conditions consisting of an initial 2‐min denaturation at 50°C and 10 min of further denaturation at 95°C, followed by 40 cycles of 15 s of denaturation at 95°C and 60 s of annealing/extension at 60°C. SYBRGreen gene expression assays were used to detect the following cytokines: pro‐inflammatory (TNF‐α and IL‐1α) and anti‐inflammatory (IL‐10 and IL‐4). Sequences of primers from each gene are shown in Table 1. 18S ribosomal gene expression was used as an endogenous control of the method. The −2^*∆∆*^ *CT* method was used to calculate the relative differences between the experimental conditions and the control groups as fold change in gene expression from real‐time quantitative PCR assays (Livak and Schmittgen 2001).

**TABLE 1:** Sequence of the forward and reverse primers used for RT‐PCR.

Gene	Forward (5′–3′)	Reverse (5′–3′)
TNF‐α	TCT CGA ACC CCG AGT GAC AA	TAT CTC TCA GCT CCA CGC CA
IL‐1α	ATG GCC AAA GTT CCA GAC ATG	TTG GTC TTC ATC TTG GGC AGT CAC
IL‐10	TCA GGG TGG CGA CTC TAT	TGG GCT TCT TC TAA ATC GTT C
IL‐4	TCA TTT TCC CTC GGT TTC AG	AGA ACA GAG GG GGA AGC AGT
18S rRNA	CGG CTA CCA CAT CCA AGG AA	GCT GGA ATT ACC GCG GCT

### 2.4 Synbiotic Yogurt Manufacturing

The yogurts were manufactured using semi‐skimmed cow's milk (1.55 g of fat, 4.65 g of sugars, 3.15 g of proteins, 0.1 g of salt, and 120 mg of calcium per 100 mL of milk; Central Lechera Asturiana, Siero, Spain). To initiate yogurt production, overnight cultures of the strains in MRS broth were centrifuged (18,500× *g* at 4°C for 15 min), and the biomass was washed three times with sterile saline solution (0.95% NaCl w/v). Then, the pellets were resuspended in the same volume of milk as the original culture and incubated at 30°C for 24 h. Simultaneously, a culture of the commercial starter Ferlac yogurt Type I in milk (Abiasa, S.L., Pontevedra, Spain), containing the species *Streptococcus thermophilus* and *Lact. delbrueckii* (1:1), was prepared following the manufacturer's instructions and was incubated at 30°C for 16 h.

For each strain, two batches of yogurt were prepared by using 50 mL of sterile, transparent yogurt cups. In one of them, 30 mL of milk supplemented with the prebiotic compound (2% v/v) was inoculated (2% v/v) with a culture of the assayed strain, prepared as mentioned above. The other was a batch‐control containing only supplemented milk. Both were incubated at 30°C for 6 h, and after this period, they were inoculated (1% v/v) with the culture of the commercial starter, and the cups were again incubated at 42°C for 6 h.

The yogurt fermentation process was monitored by pH measurement using a pH meter (Crison, Barcelona, Spain) and by plate counts using the spread‐plate method on MRS agar plates. Both analyses were performed in triplicate at different times: before strain inoculation, after strain inoculation (time 0), after 6 h of fermentation, immediately after the addition of the commercial starter, 3 h after the addition of the commercial starter, and at the completion of fermentation.

#### 2.4.1 Sensory Analysis

After the fermentation process, a sensory ranking test was conducted in accordance with the UNE‐ISO 8587:2006 standard for evaluating the attributes of odor, flavor, and appearance. The panel included 67 consumers (35 women and 32 men), ranging in age from 19 to 64 years, who were tasked with hedonically ranking the presented yogurts. Each participant assessed three different yogurt samples, which were presented in a randomized order and identified by sensory codes consisting of a letter and two numbers. The samples were served at 4°C in 50 mL transparent plastic cups.

### 2.5 Statistical Analysis

One‐way analysis of variance (ANOVA) was applied to the results, using the Student–Newman–Keuls test for comparison of the means (*p* < 0.05) in all tests to evaluate the health‐promoting properties of the strains, as well as in trials monitoring fermentation follow‐up. A post hoc test (Duncan's test) (*p* < 0.05) was applied to statistically evaluate the significant differences obtained in the prebiotic assimilation test, and Bonferroni's test (*p* < 0.05) was applied to statistically evaluate the significant differences in the gene expression assays. For the ordination test, the Friedman test was applied to determine the presence of significant differences between at least two products. Normality tests, heteroscedasticity, and independence of data were checked beforehand. Statistical analyses were performed using the IBM SPSS statistics package version 24.0 (SPSS Inc. Chicago, IL, USA).

## 3 Results and Discussion

### 3.1 Prebiotic‐Assimilation Capacity

The ability of 12 LAB strains and 2 commercial probiotic strains to assimilate 4 prebiotics (inulin, lactulose, β‐glucan, and FOS), both in their pure form and as commercial supplements, was evaluated. The results of the kinetic parameters are shown in Table S1.

Only *Lc. paracasei* UCLM‐41 and *Lp*. *plantarum* CECT 7315 were able to grow with pure inulin, and there were significant differences between them (*p* < 0.001), whereas all the strains were able to assimilate this prebiotic in the way of a commercial supplement. According to the values of the different kinetic parameters, *Lact. acidophilus* UCLM‐104 and *Lc. paracasei* UCLM‐41 stood out, since according to Duncan's test, they were the best of each group.

Pure lactulose was assimilated by all the strains, whereas with the commercial supplement, four of the LAB strains and the commercial probiotics were not able to grow. In addition, the strains had better values for the parameters μ_max_ and G with the pure prebiotic (Table S1B). Those with the most outstanding results, according to Duncan's test, were *Lp. plantarum* UCLM‐76, UCLM‐72, UCLM‐93, *Lc. paracasei* UCLM‐41, and *Lact. delbrueckii* UCLM‐32.

Pure β‐glucan was assimilated by all the LAB strains, except for *Lp. plantarum* UCLM‐36, UCLM‐37, and the commercial probiotics, while the commercial supplement was not assimilated by any of the strains. The strains, *Lp. plantarum* UCLM‐107 and *Lv. brevis* UCLM‐111, had the highest growth rate and OD_max_–OD_ini_ values, with no statistically significant differences (*p* < 0.001) between them. In addition, *Lp. plantarum* UCLM‐107 presented the lowest G value, with significant differences to the rest (*p* = 0.103).

All of the strains could assimilate both pure FOS and the commercial supplement, except for *Lc. paracasei* UCLM‐41 and *Lc. paracasei* UCLM‐24, respectively. *Lp. plantarum* UCLM‐72, *Lact. acidophilus* UCLM‐104, *Lv. brevis* UCLM‐99, and *Lact. delbrueckii* UCLM‐32 stood out with pure FOS, showing the highest μ_max_ and the lowest G values, with no significant differences between them or the commercial probiotic strain, *Lp. plantarum* CECT 7315 (*p* < 0.001). With the commercial supplement, *Lact. delbrueckii* UCLM‐32 and *Lp. plantarum* UCLM‐72 stood out, with values for the kinetic parameters significantly different from the rest, both being in the groups with the best results, according to Duncan's test.

### 3.2 Health‐Promoting Properties

#### 3.2.1 Antioxidant Activity and Total Phenolic Content

Table 2 shows the results of the antioxidant activity of the CFS at different incubation times. A progressive and significant increase in the antioxidant activity was observed during the incubation of all the LAB strains and the controls (*p* < 0.05), except for the strains, *Lact. acidophilus* UCLM‐104 and *Lv. brevis* UCLM‐111, for which values for 0.5 and 4 h were not statistically different.

**TABLE 2:** Values (mean ± SD; *n* = 3) for the antioxidant activity (mm) from the CFS of the strains at the different incubation times.

Species	Strain	Incubation time (hours)		
		0.5	4	24
*Lc*. *paracasei*	UCLM‐24	3.00^c,A^ ± 0.24	4.67^b,c,d,B^ ± 0.41	8.16^b,c,C^ ± 0.47
	UCLM‐41	3.16^c,A^ ± 0.24	5.17^b,c,d,B^ ± 0.00	12.83^e,C^ ± 0.82
*Lp*. *plantarum*	UCLM‐36	2.83^c,A^ ± 0.00	4.17^b,B^ ± 0.41	8.83^b,c,C^ ± 0.00
	UCLM‐37	2.83^c,A^ ± 0.00	4.34^b,c,B^ ± 0.24	10.83^d,C^ ± 0.82
	UCLM‐72	2.50^b,c,A^ ± 0.00	5.00^b,c,d,B^ ± 0.24	13.00^e,C^ ± 0.47
	UCLM‐76	3.17^c,A^ ± 0.47	5.50^c,d,B^ ± 0.47	14.00^e,C^ ± 0.94
	UCLM‐93	2.67^b,c,A^ ± 0.24	4.83^b,c,d,B^ ± 0.00	14.67^e,C^ ± 0.00
	UCLM‐107	2.00^a,b,A^ ± 0.41	4.83^b,c,d,B^ ± 0.24	9.66^c,d,C^ ± 0.47
*Lact*. *acidophilus*	UCLM‐104	3.00^c,A^ ± 0.41	4.83^b,c,d,A^ ± 0.00	17.34^f,B^ ± 1.25
*Lact*. *delbrueckii*	UCLM‐32	3.16^c,A^ ± 0.24	4.84^b,c,d,B^ ± 0.47	10.83^d,C^ ± 0.82
*L* *v. brevis*	UCLM‐99	3.17^c,A^ ± 0.24	5.83^d,B^ ± 0.82	17.00^f,C^ ± 0.47
	UCLM‐111	1.50^a,b,A^ ± 3.00	2.33^a,A^ ± 0.47	3.66^a,B^ ± 0.47
*Lp*. *plantarum* *	299v	2.50^b,c,A^ ± 0.00	4.00^b,B^ ± 0.41	9.33^b,c,d,C^ ± 0.82
	CECT 7315	2.33^b,c,A^ ± 0.24	3.83^b,B^ ± 0.24	7.66^b,C^ ± 0.47

*Note:* ^a–f^Different letters in the same column mean significant statistical differences (*p* < 0.05) between strains for each incubation time; ^A–C^different letters in the same row mean significant statistical differences (*p* < 0.05) between incubation times for each strain.

* *Lp. plantarum* 299v (Protransitus Lp) and *Lp. plantarum* CECT 7315 (Lactoflora Protector Inmunitario).

Comparison of values for the strains at 24 h of incubation showed that *Lv. brevis* UCLM‐99 and *Lact. acidophilus* UCLM‐104 were significant as the best strains (*p* < 0.05), without significant differences between them (*p* < 0.05). None of the commercial probiotic strains stood out for their antioxidant activity at any of the incubation times.

Results from Table 2 display an important variability both for species and for strains of the same species, which is consistent with the findings of Lepecka et al. (2023). Results for 0.5 and 4 h of incubation were similar to those reported by Hanchi et al. (2022). However, at 24 h of incubation, *Lv. brevis* UCLM‐99 and *Lact. acidophilus* UCLM‐104 displayed higher values than the strain, *Lc*. *rhamnosus* E18, from the same authors.

**FIGURE 1:**
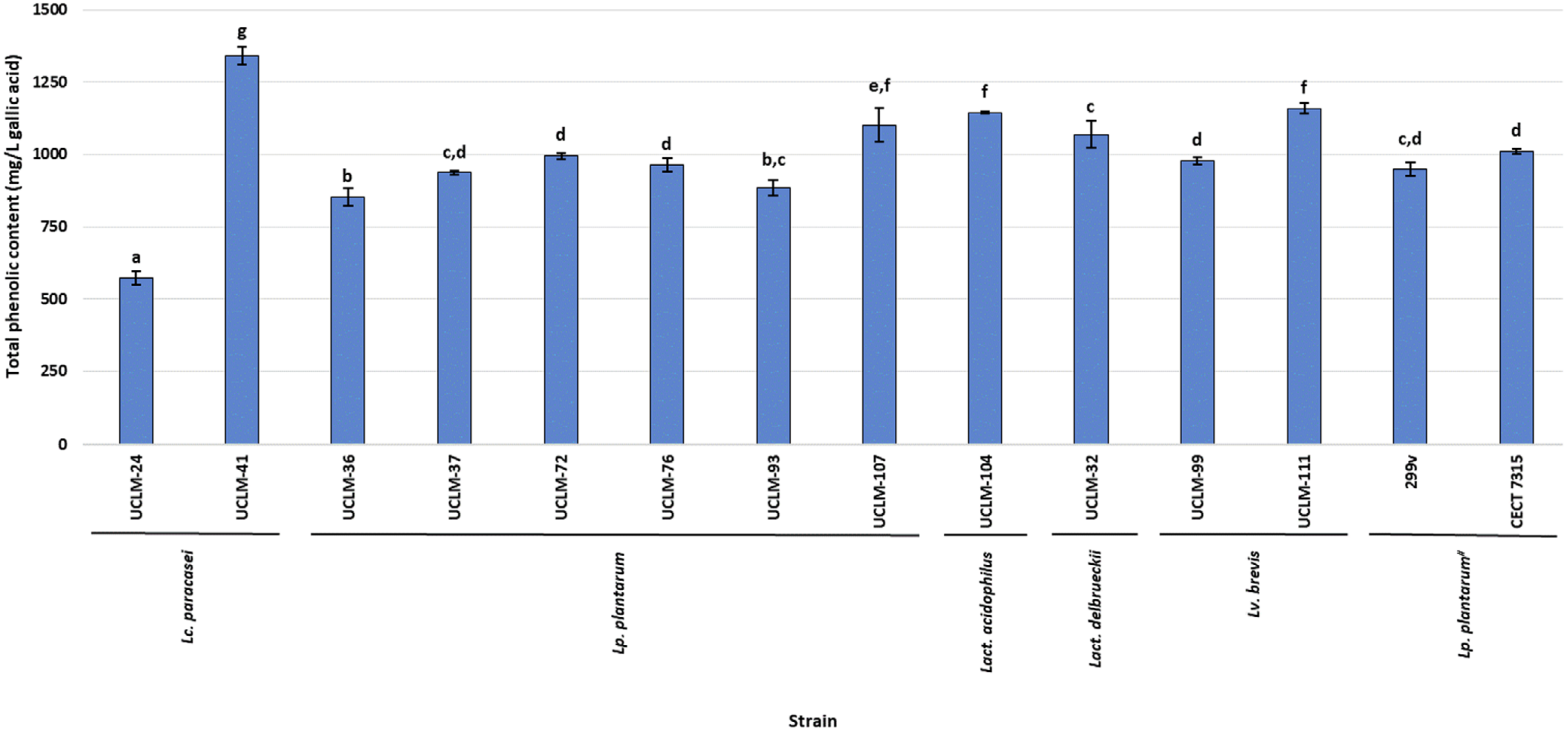
Values (mean ± SD; *n* = 3) for the total phenolic content (mg/mL) from the CFS of the strains. #: *Lp. plantarum* 299v (Protransitus Lp) and *Lp. plantarum* CECT 7315 (Lactoflora Protector Inmunitario). ^a–g^Different letters mean significant statistical differences (*p* < 0.05) between strains.

The total phenolic content of the CFS from the LAB strains (Figure 1) ranged from 573.38 mg/L of gallic acid (*Lc. paracasei* UCLM‐24) to 1340.92 mg/L of gallic acid (*Lc. paracasei* UCLM‐41), which had the highest value (*p* < 0.05). Four of the strains (*Lc. paracasei* UCLM‐41, *Lp. plantarum* UCLM‐107, *Lv. brevis* UCLM‐111, and *Lact. acidophilus* UCLM‐104) had values significantly higher than those for the probiotic commercial strains. The results in this study are lower than those in Hamad et al. (2020), but in concordance with these authors, differences for strains of the same species were obtained.

Sakoui et al. (2022) reported that antioxidant activity would be a consequence of the total phenolic content in the CFS, which is a fact that has not been corroborated by our study, as the strain, *Lv. brevis* UCLM‐111, with a high total phenolic content, showed one of the lowest values for antioxidant activity. The existence of mechanisms for antioxidant activity other than phenol production could explain this result. Hamad et al. (2020) suggested that the antioxidant activity of strains may be affected by the presence of other compounds in the CFS, such as the flavonoid, which is a fact that could explain the strain, *Lp*. *plantarum* UCLM‐93, having the third highest antioxidant activity value but the third lowest phenolic content.

#### 3.2.2 α‐Glucosidase Inhibitory Activity

**FIGURE 2:**
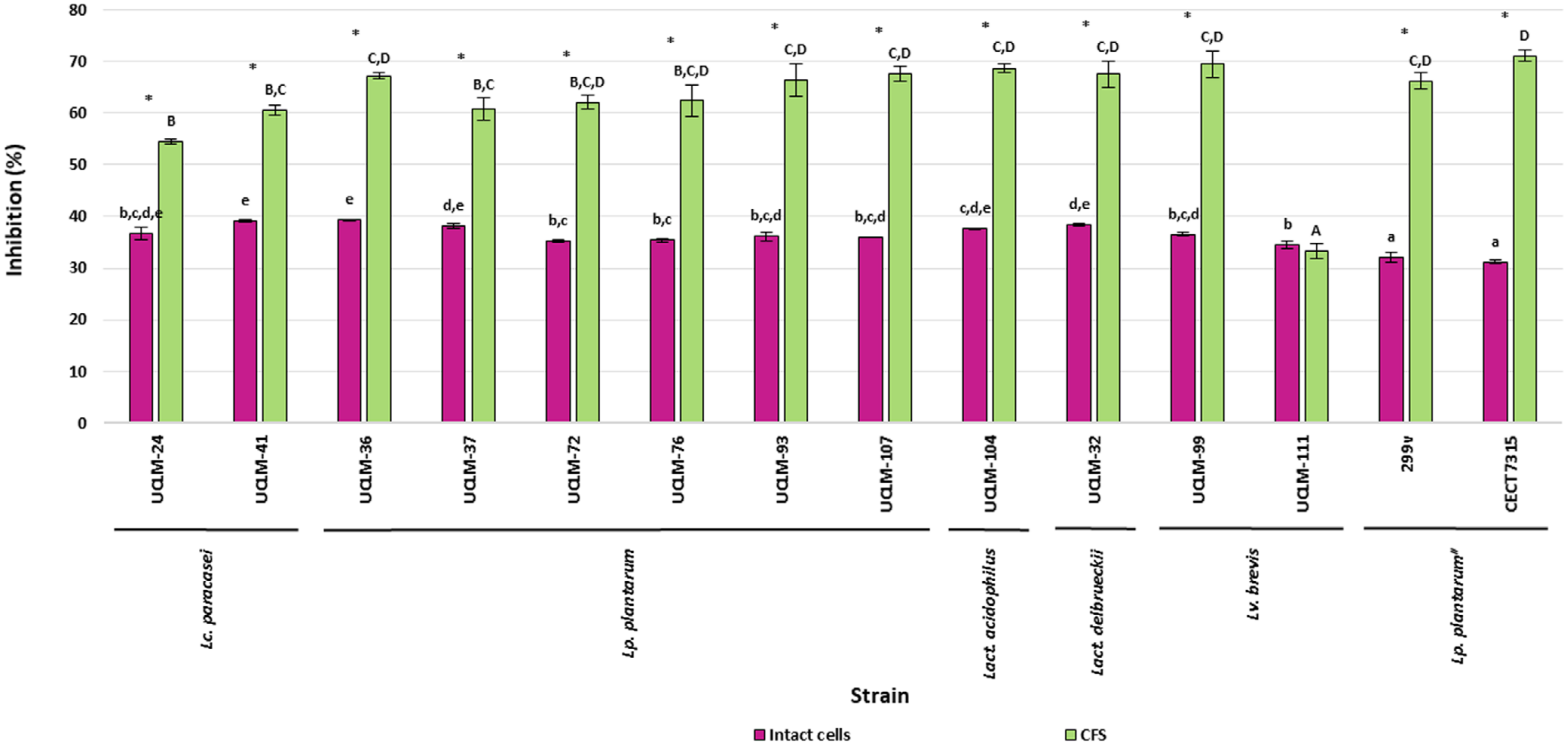
Values (mean ± SD; *n* = 3) for the inhibition of the α‐glucosidase activity (%) from intact cells and CFS of the strains. #: *Lp. plantarum* 299v (Protransitus Lp) and *Lp. plantarum* CECT 7315 (Lactoflora Protector Inmunitario). ^a–e^Different letters mean significant statistical differences (*p* < 0.05) for values from the intact cells assay. ^A–D^Different letters mean significant statistical differences (*p* < 0.05) for values from the CFS assay.

Results from both intact cells and CFS are shown in Figure 2. Values from the assay with intact cells ranged from 34.60% (*Lv. brevis* UCLM‐111) to 39.27% (*Lp. plantarum* UCLM‐36). The strains having the highest values were *Lp. plantarum* UCLM‐36 (39.27%) and *Lc. paracasei* UCLM‐41 (39.13%), which did not have statistically significant differences between them (*p* ≥ 0.05). Commercial probiotic strains had the lowest statistically significant values for α‐glucosidase inhibitory activity.

Many different values have been found in the literature for α‐glucosidase inhibitory activity from intact cells of different LAB genera. Kim et al. (2018) reported that the strain, *Lp. plantarum* K10, isolated from homemade kimchi, was able to completely inhibit (100%) α‐glucosidase activity. Lee and Kim (2019) reported a percentage of inhibition of 87% for the strain, *Leuconostoc mesenteroides* (MKSR). On the contrary, Ramos et al. (2022) reported values ranging from 17.24% to 49.16% for 40 *Leuconostoc* strains. Reuben et al. (2019) reported values for *Lactobacillus, Pediococcus*, and *Enteroccus* strains, ranging between 8.44% and 94.41%, which is a much wider range than that obtained in this study.

Values from the assay with CFS ranged from 33.32% (*Lv. brevis* UCLM‐111) to 69.42% (*Lv. brevis* UCLM‐99). Strains showing the highest values with no significant differences between them were *Lp. plantarum* UCLM‐36, UCLM‐72, UCLM‐76, UCLM‐93, and UCLM‐107, *Lact. acidophilus* UCLM‐104, *Lact. delbrueckii* UCLM‐32, and *Lv. brevis* UCLM‐99. Meanwhile, the α‐glucosidase inhibitory activity values for the CFS of the probiotic control strains, *Lp*. *plantarum* 299v and *Lp*. *plantarum* CECT 7315, were 66.19% and 71.04%, respectively. These values were not significantly different between them, nor were they different from those of the six strains showing the highest values. In concordance with the results obtained from intact cells, the strain, *Lv. brevis* UCLM‐111, showed the lowest α‐glucosidase inhibitory activity.

Comparison of the values from intact cells and CFS assays showed that for all of the LAB strains assayed, including the probiotic commercial strains, except for *Lv. brevis* UCLM‐111, the α‐glucosidase inhibitory activity was significantly higher for CFS (Figure 2), which would indicate that the compounds responsible for this activity are released by the cells into the environment. Ramchandran and Shah (2009) and Konrad et al. (2014) reported that inhibition of α‐glucosidase activity by LAB strains is related to the production of exopolysaccharides and peptides from hydrolyzed proteins, respectively.

Important differences between authors have been found, and while Zeng et al. (2016) reported lower values than those in this study for CFS of 8 *Lactobacillus* strains, Nurhayati et al. (2017) reported higher values for LAB isolates, with some strains reaching 100% inhibition. In agreement with the findings of different authors (Ramos et al. 2022; Reuben et al. 2019), the results in this study showed that α‐glucosidase inhibitory activity is a strain‐dependent property, with strains of *Lv. brevis* showing the highest and the lowest values in the CFS assay.

#### 3.2.3 Cholesterol‐Removal Ability

After 24 h of incubation, the amount of cholesterol removed by LAB ranged from 0% (*Lp. plantarum* UCLM‐93 and *Lv. brevis* UCLM‐99) to 34.51% (*Lact. acidophilus* UCLM‐104) (data not shown), with most of the strains (91.67%), including the commercial probiotic strains, having cholesterol‐removal rates lower than 20%.

In agreement with these results, Remagni et al. (2013) reported that the highest cholesterol‐removal rate in a study with 58 LAB strains of different genera was 33.1%, and Alameri et al. (2022) reported a value of 35% for two *Enterococcus faecium* isolates. Ma et al. (2019) obtained a value higher (55.2%) for the strain *Lp. plantarum* CAAS 18010.

#### 3.2.4 Peptide Content and in vitro ACE Inhibition

Table 3 shows the peptide content (mg/mL) and the value for in vitro ACE inhibition (%) in the supernatants of the milk samples after 15 h of incubation.

**TABLE 3:** Values (mean ± SD; *n* = 3) for ACE inhibitory activity (%) and peptide content (mg/mL) of the strains.

Species	Strain	Peptide content (mg/mL)	ACE inhibition (%)
*Lc*. *paracasei*	UCLM‐24	2.88^b,c^ ± 0.13	45.46^b^ ± 2.36
	UCLM‐41	3.62^d^ ± 0.04	80.23^g^ ± 0.92
*Lp*. *plantarum*	UCLM‐36	3.28^c^ ± 0.24	35.52^a^ ± 3.28
	UCLM‐37	3.10^b,c^ ± 0.05	69.48^f^ ± 0.63
	UCLM‐72	3.11^b,c^ ± 0.05	70.29^f^ ± 0.75
	UCLM‐76	1.48^a^ ± 0.07	53.58^c^ ± 2.43
	UCLM‐93	3.10^b,c^ ± 0.08	61.92^d,e^ ± 0.72
	UCLM‐107	3.63^d^ ± 0.04	45.36^b^ ± 0.82
*Lact*. *acidophilus*	UCLM‐104	3.19^b,c^ ± 0.33	60.09^d^ ± 0.25
*Lact*. *delbrueckii*	UCLM‐32	3.20^b,c^ ± 0.05	62.30^d,e^ ± 1.09
*Lv*. *brevis*	UCLM‐99	3.06^b,c^ ± 0.05	63.14^d,e^ ± 1.07
	UCLM‐111	3.77^d^ ± 0.08	55.33^c^ ± 0.13
*Lp*. *plantarum* *	299v	2.78^b^ ± 0.08	63.15^d,e^ ± 0.51
	CECT 7315	2.88^b,c^ ± 0.10	67. 51^e,f^ ± 0.30
*Lactococcus lactis* subsp. *lactis* *	A0W5	3.02^b,c^ ± 0.06	81.10^g^ ± 0.22

*Note:* ^a–g^Different letters in the same column mean significant statistical differences (*p* < 0.05) between strains.

* *Lp. plantarum* CECT 7315 (Lactoflora Protector Inmunitario) and *Lp. plantarum* 299v (Protransitus Lp).

Eighty‐three percent of the LAB strains assayed showed a peptide content higher than 3 mg/mL (Table 3). The strains, *Lc*. *paracasei* UCLM‐41, *Lp*. *plantarum* UCLM‐107, and *Lv*. *brevis* UCLM‐111, significantly showed the highest peptide content without significant differences between them, and with values higher than that for *Lactococcus lactis* subsp. *lactis* A0W5, a strain used as a control for its high proteolytic activity. *Lp*. *plantarum* UCLM‐76 showed the lowest peptide content of all the LAB strains (1.48 mg/mL), while the values for the commercial probiotic strains, *Lp. plantarum* CECT 7315 and 299v, were 2.88 and 2.78 mg/mL, respectively.

in vitro ACE inhibition by LAB strains ranged from 35.52% (*Lp. plantarum* UCLM‐36) to 80.23% (*Lc. paracasei* UCLM‐41), a value that was not significantly different of that for the strain, *Lactococcus lactis* subsp. *lactis* A0W5 (81.10%). Commercial probiotic strains inhibited ACE activity by less than 70%. The results from this study are higher than those reported by Rubak et al. (2020) for the strain, *Lact*. *kefiri* YK4 (57.36%), and by Wu, Xu, et al. (2019) for the strain, *Lact. delbrueckii* QS306 (75.58%).

When looking for a relationship between results from the peptide content and the in vitro ACE‐inhibitory activity of the strains (Table 3), it was found that it did not exist for all of the strains. Therefore, while the results from both assays, the peptide content and the in vitro ACE‐inhibitory activity, were the highest for *Lc. paracasei* UCLM‐41, this was not seen for *Lp. plantarum* UCLM‐107, as it had one of the highest values for peptide content and one of the lowest values for the in vitro ACE‐inhibitory activity. On the contrary, for the strain, *Lp. plantarum* UCLM‐76, which had the lowest peptide content, the ACE‐inhibitory activity value was higher than that for *Lp. plantarum* UCLM‐107. Zambrano‐Cervantes et al. (2023) affirmed that ACE‐inhibitory activity is not only dependent on the peptide content of the strains, but also on their different activity, which ultimately depends on their amino acidic sequence. In this respect, Rodríguez‐Figueroa et al. (2010) reported that different ACE‐inhibitory peptides are produced during the hydrolysis of milk proteins, depending on the proteolytic enzymes produced by the different strains. It has also been found that the LAB strain used is one of the major factors influencing the sequence and activity of the bioactive peptides (Rai et al. 2015).

### 3.3 Selection of the LAB Strains and the Prebiotic for the Manufacturing of Synbiotic Yogurts

A statistical analysis (Duncan's test) of the above‐mentioned results was applied to select the best LAB strains to be used for the following assays.

For antioxidant activity, the strain *Lc*. *paracasei* UCLM‐41 was in the group with the best results at 0.5 and 4 h of incubation, while the strain *Lact*. *acidophilus* UCLM‐104 had the highest value at 24 h of incubation, but this was not significantly different of the value for the strain *Lv*. *brevis* UCLM‐99. The strain *Lc*. *paracasei* UCLM‐41 had the highest phenolic content, followed by the strains, *Lact*. *acidophilus* UCLM‐104 and *Lv*. *brevis* UCLM‐111. Furthermore, the strain *Lact*. *acidophilus* UCLM‐104 was highlighted for its capacity to remove cholesterol. Regarding the α‐glucosidase inhibitory activity, both the strains *Lc*. *paracasei* UCLM‐41 and *Lact*. *acidophilus* UCLM‐104 were in the group with the best values when intact cells were assayed, while the strain *Lact*. *acidophilus* UCLM‐104 was in the best group when CFS was assayed. Finally, the strain *Lc. paracasei* UCLM‐41 was the strain that most inhibited ACE activity. In consideration of these results, the strains *Lc*. *paracasei* UCLM‐41 and *Lact*. *acidophilus* UCLM‐104 were selected to produce the synbiotic yogurt. Therefore, we only considered the results of the assimilation capacity assay from these strains for selecting the prebiotic to add to milk for the manufacturing of the synbiotic yogurts.

**FIGURE 3:**
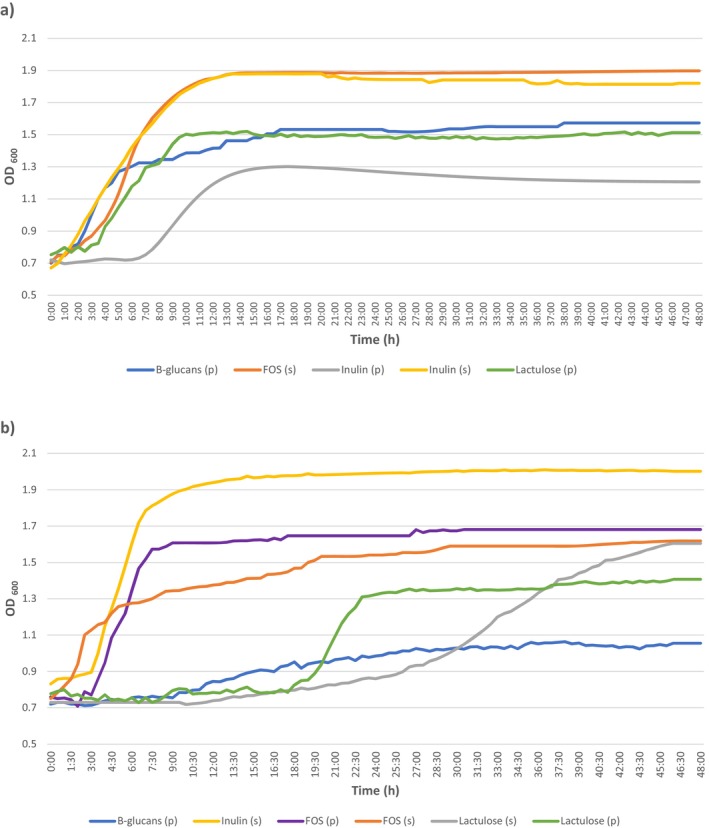
Growth curves for the strains *Lc. paracasei* UCLM‐41 (a) and *Lact. acidophilus* UCLM‐104 (b) using MRS broth added with different prebiotics, both as a pure compound (p) or as a commercial supplement (s).

Figure 3 shows the growth curves of the strains *Lc*. *paracasei* UCLM‐41 (a) and *Lact*. *acidophilus* UCLM‐104 (b), with the different prebiotics, and it can be observed that both strains grew the highest with the commercial supplement of inulin. Therefore, it was selected for the synbiotic yogurt production, though *Lc*. *paracasei* UCLM‐41 did the same with the commercial supplement of FOS.

### 3.4 Immunomodulatory Activity Assay

An assay to determine the anti‐inflammatory effect of the selected strains, *Lact. acidophilus* UCLM‐104 and *Lc. paracasei* UCLM‐41, on LPS‐induced inflammation in Caco‐2 cells was carried out by measurement of mRNA levels of pro‐inflammatory (TNF‐α and IL‐1α) and anti‐inflammatory (IL‐10 and IL‐4) cytokines.

**FIGURE 4:**
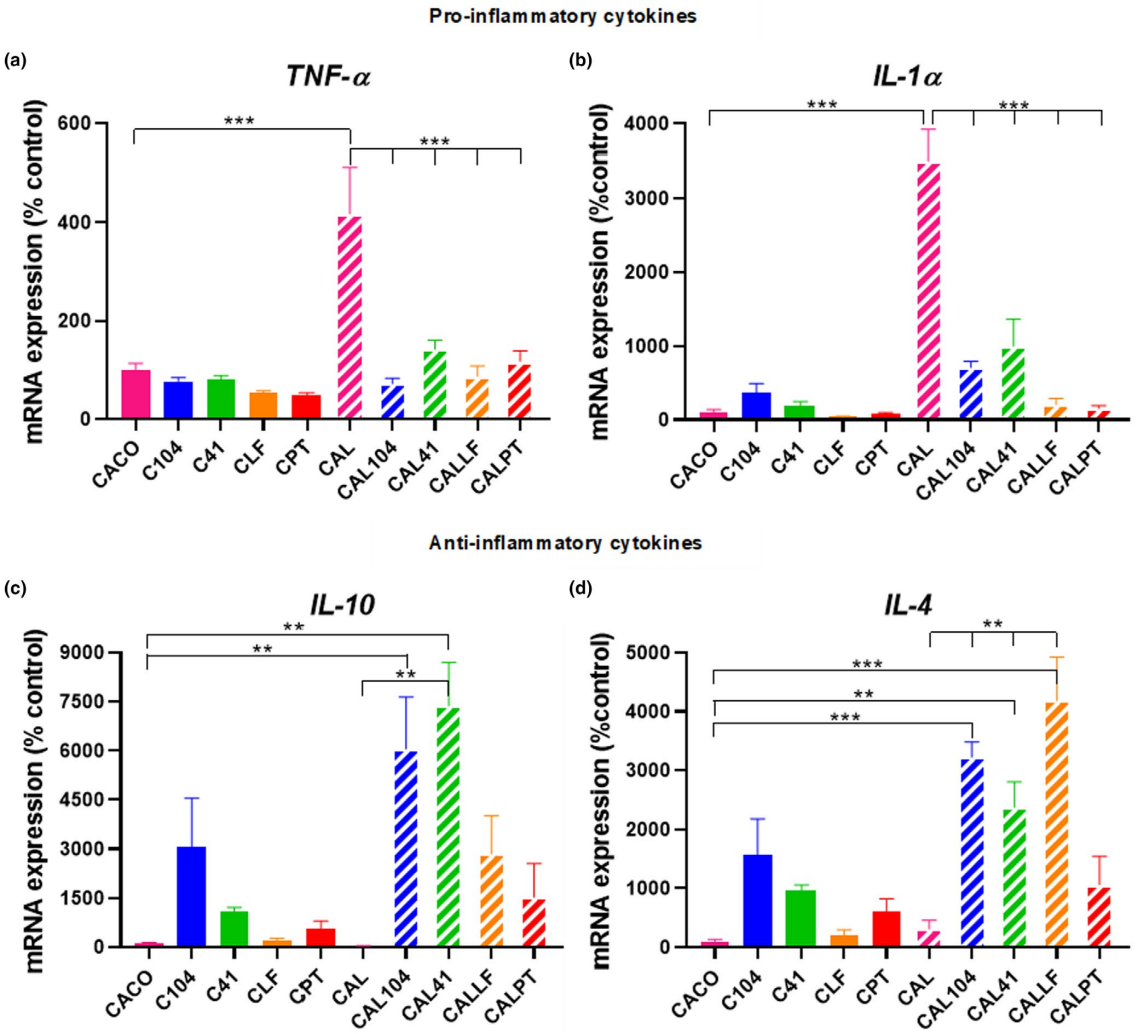
Relative mRNA levels (mean ± SD; *n* = 4) of pro‐inflammatory cytokines TNF‐α (a) and IL‐1α (b) and anti‐inflammatory cytokines IL‐10 (c) and IL‐4 (d) in Caco‐2 cell cultures, respectively. On one hand, LAB strains *Lact. acidophilus* UCLM‐104 (C104), *Lc. paracasei* UCLM‐41 (C41), and commercial probiotic strains *Lp. plantarum* CECT 7315 (CLF) and *Lp. plantarum* 299v (CPT) were added to the LPS‐untreated Caco‐2 cell cultures (CACO). On the other hand, LAB strains *Lact. acidophilus* UCLM‐104 (CAL104), *Lc. paracasei* UCLM‐41 (CAL41), and commercial probiotic strains *Lp. plantarum* CECT 7315 (CALLF) and *Lp. plantarum* 299v (CALPT) were added to the LPS‐treated Caco‐2 cell cultures (CAL). *p < 0.05, **p < 0.01, and ***p < 0.001 compared to CACO or CAL.

As shown in Figure 4a,b, LPS significantly increased the mRNA levels of TNF‐α and IL‐1α in Caco‐2 cells (*p* < 0.05), whereas the exposure to selected LAB strains or commercial probiotic strains significantly reduced this increase in LPS‐treated Caco‐2 cells, as they achieved results similar to previous values in LPS‐induced inflammation, without significant differences between the strains (*p* ≥ 0.05). However, the addition of selected LAB strains notably increased the mRNA levels of the anti‐inflammatory cytokine IL‐10 in LPS‐treated Caco‐2 cells, with significant differences when the strains *Lact*. *acidophilus* UCLM‐104 or *Lc*. *paracasei* UCLM‐41 were added to the culture (*p* < 0.05) (Figure 4c). The same effect was observed in the case of the other anti‐inflammatory cytokine assayed, IL‐4, where its expression was significantly increased when the strains *Lact*. *acidophilus* UCLM‐104, *Lc*. *paracasei* UCLM‐41, or *Lp*. *plantarum* CECT 7315 (LF) were added to the LPS‐treated cultures (*p* < 0.05). Furthermore, the addition of LAB strains or commercial probiotic strains induced a slight augment in LPS‐untreated Caco‐2 cells, as this effect was more prominent in the case of the LAB strains, although there were no significant differences in any case (*p* ≥ 0.05) (Figure 4d). Therefore, these findings demonstrate that the strains *Lact. acidophilus* UCLM‐104 and *Lc. paracasei* UCLM‐41 modulate LPS‐induced inflammation in intestinal cells, decreasing the expression of pro‐inflammatory mediators and boosting the anti‐inflammatory mediators.

According to Mazziotta et al. (2023), probiotics can either directly or indirectly influence the adaptive immune response and establish a network of signals among the different immune cells by stimulating the production of cytokines, including ILs, TNFs, and chemokines, by either immune cells, such as lymphocytes or macrophages, or by intestinal epithelial cells. Anti‐inflammatory cytokines acting as immunoregulatory molecules, such as IL‐10, can control pro‐inflammatory cytokine responses to maintain gut homeostasis (Mazziotta et al. 2023). Specific pro‐inflammatory cytokines involved in intestinal inflammation include IFN‐γ, IL‐1α, and TNF‐α, thus, a decrease in the production of these cytokines contributes to the improvement of inflammatory symptoms (Mazziotta et al. 2023).

Here, we report that potential probiotic LAB strains can reduce the expression of pro‐inflammatory cytokines in vitro, as well as increase the expression of anti‐inflammatory cytokines, which is in accordance with the results found in previous literature (Bai and Ouyang 2006; Lindfors et al. 2008). Bai and Ouyang (2006) stated that a significant decrease in pro‐inflammatory cytokine TNF‐α expression was seen in co‐cultures of intestinal mucosa from Crohn's disease patients with *Lact*. *casei*, *Lact*. *bulgaricus*, and *Lact*. c*rispatus*. Lindfors et al. (2008) showed that certain probiotics, such as *Lact*. *fermentum* and *Bifidobacterium lactis* , stimulate the production of IL‐10 by T‐regulatory cells in cultures of Caco‐2 cells. Probiotics interact with immunocompetent cells using the mucosal interface and locally modulate the production of pro‐ and anti‐inflammatory cytokines; thus, they can have potential therapeutic effects on inflammatory bowel diseases such as Crohn's disease or celiac disease (Rai et al. 2015).

### 3.5 Synbiotic Yogurt Manufacturing

For the yogurt manufacturing, the selected strains, *Lc. paracasei* UCLM‐41 and *Lact. acidophilus* UCLM‐104, were inoculated (2%) together with a commercial starter in semi‐skimmed cow's milk supplemented with the commercial supplement of inulin (2% w/v). In parallel, a control yogurt was prepared with the same milk, but was only inoculated with the commercial starter. The fermentation process was followed by pH measurement and viable cell counts (Figure S1).

Throughout the fermentation process, no significant differences in the pH values were observed between yogurts made with the selected strains, but each was significantly different (*p* < 0.05) compared to the control yogurt manufactured without the addition of any probiotic bacteria. Therefore, while the control yogurt reached a pH value of 5.1 after 6 h of inoculation, yogurts made with the probiotic bacteria, *Lact*. *acidophilus* UCLM‐104 or *Lc*. *paracasei* UCLM‐41, reached pH values of 4.2 and 4.1, respectively.

The initial population of probiotic bacteria at both batches was 8‐log cfu/mL, and both followed the same evolution for the viable cell counts throughout the process, without significant differences between them (*p* ≥ 0.05) (Figure S1). Control yogurts, that were inoculated with 8‐log cfu/mL at 6 h, reached the same population at the end of fermentation as those inoculated with the potential probiotic strains (11‐log cfu/mL). The synbiotic yogurts met the minimum requirement of 7–8‐log cfu/mL of probiotic product required to provide health benefits to consumers (Mohammadi et al. 2012).

#### 3.5.1 Sensory Analysis

The experimental F (F_exp_) value for the attributes odor, flavor, and appearance was 24.34, 34.94, and 72.75, respectively, taking into account the following: j = 67 judges; *p* = 3 samples; Rn = sum of product ordinations, and the tabulated F (F_tabulated_) = 5.99 (95% confidence level). Since the F_exp_ was greater than the F_tabulated_ in all cases, the null hypothesis was rejected with a risk of error of 5%, indicating that there were significant differences among the samples for the three attributes.

The value from the Minimum Significant Difference Test (MSD) was 22.69, at a risk level of *α* = 0.05 and Z = 1.96 (*α* = 0.05, bilateral normal probability). This demonstrated that there were no significant differences between the yogurts manufactured with each of the potential probiotic strains, *Lc. paracasei* UCLM‐41 and *Lact. acidophilus* UCLM‐104, but there were statistically significant differences between them and the control yogurts.

## 4 Conclusions

The outcomes of the current study have confirmed the strain‐dependence of many of the properties analyzed and have facilitated the selection of two probiotic LAB strains for yogurt manufacturing, namely *Lc. paracasei* UCLM‐41 and *Lact. acidophilus* UCLM‐104, characterized by compelling antioxidant, anti‐diabetic, anti‐hypertensive, and anti‐inflammatory properties. Notably, these properties surpassed those of the commercial probiotic strains utilized as controls in this research. Upon evaluating the assimilation capacity of various prebiotics, both strains exhibited the highest growth when utilizing a commercial supplement of inulin, prompting their selection for the manufacturing of synbiotic yogurts.

The synbiotic yogurts produced with *Lc. paracasei* UCLM‐41 and *Lact. acidophilus* UCLM‐104 displayed superior sensory attributes compared to the control batch, which was prepared with a conventional yogurt starter, and garnered greater acceptance from taste‐testers. The findings strongly suggest that these strains hold promise as probiotics to produce synbiotic yogurts with exceptional sensory qualities; however, additional investigations are warranted to assess if, indeed, the yogurts manufactured with them have the desired antioxidant, anti‐diabetic, hypocholesterolemic and anti‐hypertensive properties. Likewise, it would be convenient to determine the in vivo activity, using animal models, aligning with recommendations from the Food and Agriculture Organization of the United Nations (FAO)/World Health Organization (WHO) (2002).

To the best of our knowledge, this study represents a pioneering endeavor, constituting the first comprehensive assessment of the health‐promoting properties of potential probiotic strains, followed by their application in the production of synbiotic yogurts.

## Author Contributions

S.R.‐S.: validation, methodology, investigation, formal analysis, and writing – original draft; P.F.‐P.: validation, methodology, investigation, formal analysis, and writing – original draft; C.R.‐R.: validation, methodology, and investigation; E.B.‐R.: validation, methodology, investigation, formal analysis, and writing – original draft; S.S.: validation, formal analysis, writing – review and editing, and supervision; M.L.P.: conceptualization, validation, formal analysis, writing – review and editing, supervision, and project administration.

## Ethics Statement

Participants gave informed consent via the statement “I am aware that my responses are confidential, and I agree to participate in this sensory ranking test in accordance with the UNE‐ISO 8587:2006 standard”. They were able to withdraw from the sensory analysis at any time without giving a reason. The yogurts tested were safe for consumption.

## Conflicts of Interest

The authors declare no conflicts of interest.

## Supporting information


**Figure S1.** Values (mean; *n* = 3) for viable cell counts (log cfu/mL) and for the pH during the manufacture of the yogurt. *: value before inoculation of the commercial starter. **: value immediately after inoculation of the starter.

**Table S1.** Values (mean ± SD; *n* = 3) for the kinetic parameters from strains growing with (A) inulin, (B) lactulose, (C) β‐glucans and (D) FOS, both in its pure form (p) and as a commercial supplement(s).

## Data Availability

The data that support the findings of this study are available from the corresponding author upon reasonable request.
