# Supplementary figures and images for: Lactobacillus acidophilus UCLM‐104 and Lacticaseibacillus paracasei UCLM‐41 Are Promising Candidates to Produce Synbiotic Yogurt
Source: Food Sci Nutr. 2025 Jun 30;13(7):e70539. doi: 10.1002/fsn3.70539 (PMC12208914; doi:10.1002/fsn3.70539)

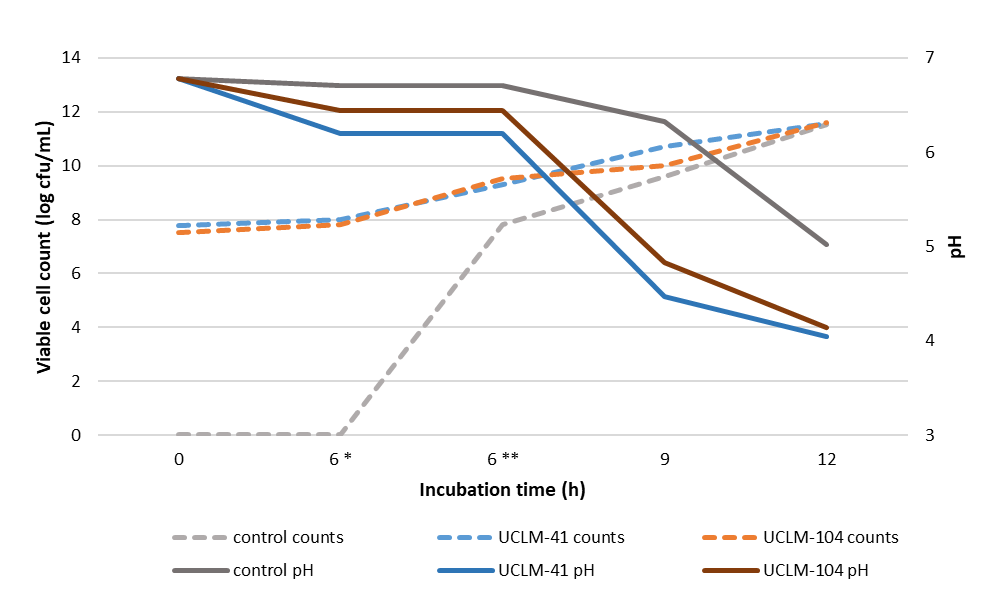
**Fig. S1**

Supplement: Supplementary file 1 — Figure S1. Values (mean; n = 3) for viable cell counts (log cfu/mL) and for the pH during the manufacture of the yogurt. *: value before inoculation of the commercial starter. **: value immediately after inoculation of the starter. [file FSN3-13-e70539-s002.docx]
