# Supplementary material for: Lactobacillus acidophilus UCLM‐104 and Lacticaseibacillus paracasei UCLM‐41 Are Promising Candidates to Produce Synbiotic Yogurt
Source: Food Sci Nutr. 2025 Jun 30;13(7):e70539. doi: 10.1002/fsn3.70539 (PMC12208914; doi:10.1002/fsn3.70539)
Supplement: Supplementary file 2 — Table S1. Values (mean ± SD; n = 3) for the kinetic parameters from strains growing with (A) inulin, (B) lactulose, (C) β‐glucans and (D) FOS, both in its pure form (p) and as a commercial supplement(s). [file FSN3-13-e70539-s001.docx]

**Table S1** Values (mean ± SD; n = 3) for the kinetic parameters from strains growing with (A) inulin, (B) lactulose, (C) β-glucans and (D) FOS, both in its pure form (p) and as a commercial supplement (s)

(A)

| **Species** | **Strain** | **Inulin** | **Kinetic parameters** | | | | | | | |
| --- | --- | --- | --- | --- | --- | --- | --- | --- | --- | --- |
|  |  |  | **µ_max_ (h^-1^)** | | **G (h)** | | **Δ OD_max_-OD_ini_ (600nm)** | | **λ (h)** | |
|  |  |  |  | **Sig** |  | **Sig** |  | **Sig** |  | **Sig** |
| ***Lc*. *paracasei*** | **UCLM-24** | p | - |  | - |  | - |  | - |  |
|  |  | s | 0.05 ± 0.02^u,v,w,x^ |  | 7.60 ± 3.50^t,u,v,w,x^ |  | 0.79 ± 0.26^v,w^ |  | 0.66 ± 0.02^t,u^ |  |
|  | **UCLM-41** | p | 0.04 ± 0.00^v,w,x,y^ | * | 6.95 ± 0.50^u,v^ | * | 0.63 ± 0.07^w,x^ | * | 0,70 ± 0.42 | ns |
|  |  | s | 0.06 ± 0.00^t,u^ |  | 4.99 ± 0.26^t,u^ |  | 1.23 ± 0.02^t^ |  | 0,69 ± 0.021^t,u,v^ |  |
| ***Lp*. *plantarum*** | **UCLM-36** | p | - |  | - |  | - |  | - |  |
|  |  | s | 0.05 ± 0.03^u,v,w^ |  | 9.50 ± 7.90^t,u,v,w,x,y^ |  | 0.89 ± 0.17^u,v,x^ |  | 0.73 ± 0.04^u,v,w,x^ |  |
|  | **UCLM-37** | p | - |  |  |  |  |  |  |  |
|  |  | s | 0.03 ± 0.01^x,y^ |  | 11.00 ± 2.50^w,x,y^ |  | 0.50 ± 0.11^x,y^ |  | 0.72 ± 0.01^u,v,w,x^ |  |
|  | **UCLM-72** | p | - |  | - |  | - |  | - |  |
|  |  | s | 0.03 ± 0.01^x,y^ |  | 11.20 ± 2.10^w,x,y^ |  | 0.69 ± 0.12^w,x^ |  | 0.70 ± 0.07^u,v,w^ |  |
|  | **UCLM-76** | p | - |  | - |  | - |  | - |  |
|  |  | s | 0.03 ± 0.00^w,x,y^ |  | 9.90 ± 1.50^u,v,w,x,y^ |  | 0.98 ± 0.08^u,v^ |  | 0.79 ± 0.06^x^ |  |
|  | **UCLM- 93** | p | - |  | - |  | - |  | - |  |
|  |  | s | 0.02 ± 0.00^y^ |  | 13.00 ± 2.60^y^ |  | 0.40 ± 0.08^y^ |  | 0.62 ± 0.03^t^ |  |
|  | **UCLM-107** | p | - |  | - |  | - |  | - |  |
|  |  | s | 0.05 ± 0.00^t,u,v^ |  | 5.59 ± 0.57^t,u,v^ |  | 0.94 ± 0.05^u,v^ |  | 0.74 ± 0.04^v,w,x^ |  |
| ***Lb***. ***acidophilus*** | **UCLM-104** | p | - |  | - |  | - |  | - |  |
|  |  | s | 0.07 ± 0.00^t,u^ |  | 4.61 ± 2.40^v,w,x,y^ |  | 1.20 ± 0.10^t,u^ |  | 0.82 ± 0.03^x^ |  |
| ***Lb*. *delbrueckii*** | **UCLM-32** | p | - |  | - |  | - |  | - |  |
|  |  | s | 0.05 ± 0.00^u,v^ |  | 5.73 ± 0.15^t,u,v^ |  | 1.01 ± 0.16^u^ |  | 0.72 ± 0.03^u,v,w,x^ |  |
| ***L*. *brevis*** | **UCLM-99** | p | - |  | - |  | - |  | - |  |
|  |  | s | 0.04 ± 0.01^v,w,x,y^ |  | 8.30 ± 1.50^t,u,v,w,x,y^ |  | 0.91 ± 0.08^u,v^ |  | 0.80 ± 0.04^x^ |  |
|  | **UCLM-111** | p | - |  | - |  | - |  | - |  |
|  |  | s | 0.05 ± 0.00^u,v,w^ |  | 6.35 ± 0.34^t,u,v,w^ |  | 0.68 ± 0.04^w,x^ |  | 0.66 ± 0.01^t,u^ |  |
| ***Lp*. *plantarum*^#^** | **299v** | p | - |  | - |  | - |  | - |  |
|  |  | s | 0.03 ± 0.00^y^ |  | 12.24 ± 0.13^x,y^ |  | 0.54 ± 0.02^x,y^ |  | 0.75 ± 0.01^v,w,x^ |  |
|  | **CECT 7315** | p | 0.05 ± 0.01^u,v,w^ | ns | 6.17 ± 0.73^u,v^ | * | 0.96 ± 0.68^w,x^ | ns | 0.92 ± 0.61^x^ | ns |
|  |  | s | 0.07 ± 0.00^t^ |  | 4.30 ± 0.16^t^ |  | 1.06 ± 0.03^t,u^ |  | 0.75 ± 0.04^v,w,x^ |  |

(B)

| **Species** | **Strain** | **Lactulose** | **Kinetic parameters** | | | | | | | |
| --- | --- | --- | --- | --- | --- | --- | --- | --- | --- | --- |
|  |  |  | **µ_max_ (h^-1^)** | | **G (h)** | | **Δ OD_max_-OD_ini_ (600nm)** | | **λ (h)** | |
|  |  |  |  | **Sig** |  | **Sig** |  | **Sig** |  | **Sig** |
| ***Lc*. *paracasei*** | **UCLM-24** | p | 0.05 ± 0.00^b^ |  | 5.70 ± 0.20^a,b^ |  | 1.00 ± 0.19^b,c^ |  | 0.76 ± 0.07^a.b^ |  |
|  |  | s | - |  | - |  | - |  | - |  |
|  | **UCLM-41** | p | 0.06 ± 0.01^b^ |  | 5.70 ± 1.30^a,b^ |  | 1.18 ± 0.35^a,b,c^ |  | 0.88 ± 0.06^b,c,d^ |  |
|  |  | s | - |  | - |  | - |  | - |  |
| ***Lp*. *plantarum*** | **UCLM-36** | p | 0.05 ± 0.01^b^ |  | 6.00 ± 1.40^a,b^ |  | 1.32 ± 0.07^a^ |  | 0.96 ± 0.05^c,d^ |  |
|  |  | s | - |  | - |  | - |  | - |  |
|  | **UCLM-37** | p | 0.05 ± 0.00^b^ | * | 5.80 ± 0.27^a,b^ | * | 1.07 ± 0.11^a,b,c^ | ns | 0.99 ± 0.11^c,d^ | ns |
|  |  | s | 0.02 ± 0.00^u,v^ |  | 14.51 ± 0.36^v^ |  | 1.11 ± 0.02^t,u^ |  | 0.97 ± 0.01^w,x^ |  |
|  | **UCLM-72** | p | 0.06 ± 0.01^a,b^ | * | 5.10 ± 0.55^a,b^ | * | 1.10 ± 0.13^a,b,c^ | ns | 0.85 ± 0.05^b,c^ | ns |
|  |  | s | 0.03 ± 0.00^t^ |  | 10.37 ± 0.32^t^ |  | 0.94 ± 0.04^v^ |  | 0.91 ± 0.02^w^ |  |
|  | **UCLM-76** | p | 0.06 ± 0.01^a,b^ | * | 5.10 ± 0.72^a,b^ | * | 1.20 ± 0.07^a,b,c^ | * | 0.83 ± 0.07^a,b,c^ | ns |
|  |  | s | 0.02 ± 0.00^u,v^ |  | 14.47 ± 0.43^v^ |  | 0.72 ± 0.06^w^ |  | 0.82 ± 0.03^v^ |  |
|  | **UCLM- 93** | p | 0.05 ± 0.00^b^ | * | 5.60 ± 0.39^a,b^ | * | 1.01 ± 0.08^a,b,c^ | * | 0.82 ± 0.10^a,b,c^ | ns |
|  |  | s | 0.02 ± 0.00^v^ |  | 16.47 ± 0.39^w^ |  | 0.61 ± 0.04^x^ |  | 0.74 ± 0.05^u^ |  |
|  | **UCLM-107** | p | 0.07 ± 0.01^a^ | * | 4.31 ± 0.60^a^ | * | 1.11 ± 0.09^a,b,c^ | ns | 0.98 ± 0.13^c,d^ | * |
|  |  | s | 0.03 ± 0.00^t^ |  | 11.10 ± 1.10^t,u^ |  | 1.10 ± 0.03^u^ |  | 0.60 ± 0.01^t^ |  |
| ***Lb***. ***acidophilus*** | **UCLM-104** | p | 0.05 ± 0.00^b^ | * | 5.78 ± 0.49^a,b^ | * | 0.90 ± 0.20^c^ | ns | 0.85 ± 0.15^b,c^ | ns |
|  |  | s | 0.02 ± 0.00^u^ |  | 13.30 ± 2.40^v^ |  | 1.07 ± 0.09^u^ |  | 1.03 ± 0.04^x^ |  |
| ***Lb*. *delbrueckii*** | **UCLM-32** | p | 0.05 ± 0.01^b^ | * | 5.90 ± 1.30^a,b^ | * | 1.24 ± 0.11^a,b^ | * | 0.92 ± 0.06^b,c,d^ | ns |
|  |  | s | 0.02 ± 0.00^u,v^ |  | 14.00 ± 0.85^v^ |  | 1.03 ± 0.05^u,v^ |  | 0.91 ± 0.02^w^ |  |
| ***L*. *brevis*** | **UCLM-99** | p | 0.05 ± 0.01^b^ | * | 5.80 ± 1.20^a,b^ | * | 0.98 ± 0.17^b,c^ | ns | 0.92 ± 0.16^b,c,d^ | ns |
|  |  | s | 0.02 ± 0.00^u^ |  | 12.79 ± 0.53^u,v^ |  | 1.19 ± 0.07^t^ |  | 1.10 ± 0.07^y^ |  |
|  | **UCLM-111** | p | 0.05 ± 0.01^b,c^ |  | 6.80 ± 1.10^b^ |  | 1.08 ± 0.19^a,b,c^ |  | 1.04 ± 0.12^d^ |  |
|  |  | s | - |  | - |  | - |  | - |  |
| ***Lp*. *plantarum*^#^** | **299v** | p | 0.03 ± 0.01^c^ |  | 9.20 ± 1.5^c^ |  | 1.05 ± 0.14^a,b,c^ |  | 0.91 ± 0.01^b,c,d^ |  |
|  |  | s | - |  | - |  | - |  | - |  |
|  | **CECT 7315** | p | 0.07 ± 0.00^a^ |  | 4.25 ± 0.03^a^ |  | 0.93 ± 0.02^b,c^ |  | 0.66 ± 0.01^a^ |  |
|  |  | s | - |  | - |  | - |  | - |  |

(C)

| **Species** | **Strain** | **β-glucans** | **Kinetic parameters** | | | |
| --- | --- | --- | --- | --- | --- | --- |
|  |  |  | **µ_max_ (h^-1^)** | **G (h)** | **Δ OD_max_-OD_ini_ (600nm)** | **λ (h)** |
| ***Lc. paracasei*** | **UCLM-24** | p | 0.03 ± 0.00^b,c,d^ | 11.5 ± 2.10^a,b^ | 0.47 ± 0.04^c^ | 0.63 ± 0.11^a^ |
|  | **UCLM-41** | p | 0.02 ± 0.00^c,d,e^ | 20.10 ± 0.00^a,b^ | 0.50 ± 0.00^c^ | 1.10 ± 0.0^b^ |
| ***Lp. plantarum*** | **UCLM-36** | p | - | - | - | - |
|  | **UCLM-37** | p | - | - | - | - |
|  | **UCLM-72** | p | 0.03 ± 0.01^b,c^ | 12.40 ± 6.30^a,b^ | 0.46 ± 0.15^c^ | 0.66 ± 0.27^a^ |
|  | **UCLM-76** | p | 0.03 ± 0.01^b,c,d^ | 13.30 ± 6.90^a,b^ | 0.50 ± 0.20^c^ | 0.76 ± 0.27^a,b^ |
|  | **UCLM- 93** | p | 0.03 ± 0.01^b,c^ | 11.20 ± 2.20^a,b^ | 0.51 ± 0.08^c^ | 0.60 ± 0.14^a^ |
|  | **UCLM-107** | p | 0.05 ± 0.01^a^ | 5.8 ± 0.60^a^ | 0.93 ± 0.06^a^ | 0.58 ± 0.03^a^ |
| ***Lb. acidophilus*** | **UCLM-104** | p | 0.03 ± 0.01^b,c^ | 11.10 ± 2.40^a,b^ | 0.57 ± 0.07^c^ | 0.59 ± 0.02^a^ |
| ***Lb. delbrueckii*** | **UCLM-32** | p | 0.04 ± 0.01^b^ | 9.40 ± 3.80^a,b^ | 0.66 ± 0.09^b,c^ | 0.61 ± 0.15^a^ |
| ***L. brevis*** | **UCLM-99** | p | 0.03 ± 0.01^b,c^ | 9.90 ± 2.40^a,b^ | 0.61 ± 0.08^b,c^ | 0.66 ± 0.16^a^ |
|  | **UCLM-111** | p | 0.07 ± 0.02^a,b^ | 9.7 ± 5.6^a,b^ | 0.81 ± 0.15^a,b^ | 0.75 ± 0.25^a,b^ |
| ***Lp. plantarum^#^*** | **299v** | p | - | - | - | - |
|  | **CECT 7315** | p | - | - | - | - |

(D)

| **Species** | **Strain** | **FOS** | **Kinetic parameters** | | | | | | | |
| --- | --- | --- | --- | --- | --- | --- | --- | --- | --- | --- |
|  |  |  | **µ_max_ (h^-1^)** | | **G (h)** | | **Δ OD_max_-OD_ini_ (600nm)** | | **λ (h)** | |
|  |  |  |  | **Sig** |  | **Sig** |  | **Sig** |  | **Sig** |
| ***Lc*. *paracasei*** | **UCLM-24** | p | 0.04 ± 0.00^d^ |  | 8.50 ± 1.20^e^ |  | 0.88 ± 0.02^c,d,e,f^ |  | 0.76 ± 0.03^b,c^ |  |
|  |  | s | - |  | - |  | - |  | - |  |
|  | **UCLM-41** | p | - |  | - |  | - |  | - |  |
|  |  | s | 0.06 ± 0.00^v,w^ |  | 5.09 ± 0.21^t,u,v^ |  | 1.19 ± 0.01^t^ |  | 0.73 ± 0.01^v,w^ |  |
| ***Lp*. *plantarum*** | **UCLM-36** | p | 0.05 ± 0.01^b,c^ | * | 6.56 ± 0.77^c,d^ | * | 0.84 ± 0.18^d,e,f^ | ns | 0.64 ± 0.02^a^ | ns |
|  |  | s | 0.06 ± 0.01^u,v^ |  | 4.81 ± 0.58^t,u^ |  | 1.05 ± 0.02^u^ |  | 0.75 ± 0.03^w,x^ |  |
|  | **UCLM-37** | p | 0.04 ± 0.00^b,c^ | ns | 6.72 ± 0.27^d^ | * | 0.80 ± 0.22^d,e,f^ | ns | 0.70 ± 0.00^a,b^ | ns |
|  |  | s | 0.06 ± 0.00^x^ |  | 5.91 ± 0.43^v,w^ |  | 0.84 ± 0.06^x,y^ |  | 0.69 ± 0.01^t,u,v^ |  |
|  | **UCLM-72** | p | 0.07 ± 0.00^a^ | ns | 4.40 ± 0.11^a^ | * | 0.87 ± 0.10^c,d,e,f^ | ns | 0.70 ± 0.01^a,b^ | ns |
|  |  | s | 0.07 ± 0.00^t^ |  | 4.11 ± 0.11^t^ |  | 0.93 ± 0.05^v,w^ |  | 0.72 ± 0.01^u,v,w^ |  |
|  | **UCLM-76** | p | 0.05 ± 0.00^b^ | ns | 5.79 ± 0.43^c,d^ | ns | 0.92 ± 0.04^b,c,d,e^ | ns | 0.72 ± 0.02^a,b,c^ | * |
|  |  | s | 0.05 ± 0.00^x^ |  | 6.24 ± 0.20^w^ |  | 0.89 ± 0.01^w,x^ |  | 0.82 ± 0.03^z^ |  |
|  | **UCLM- 93** | p | 0.07 ± 0.00^a^ | * | 4.59 ± 0.08^a,b^ | * | 0.68 ± 0.01^f^ | * | 1.13 ± 0.06^d^ | * |
|  |  | s | 0.06 ± 0.00^u,v^ |  | 4.76 ± 0.02^t,u^ |  | 0.63 ± 0.01^z^ |  | 0.72 ± 0.00^u,v,w^ |  |
|  | **UCLM-107** | p | 0.04 ± 0.00^c,d^ | ns | 7.78 ± 0.46^e^ | ns | 0.80 ± 0.10^e,f^ | * | 0.74 ± 0.02^b,c^ | ns |
|  |  | s | 0.04 ± 0.01^y^ |  | 8.70 ± 1.6^x^ |  | 1.01 ± 0.04^u,v^ |  | 0.79 ± 0.04^x,y,z^ |  |
| ***Lb***. ***acidophilus*** | **UCLM-104** | p | 0.07 ± 0.01^a^ | ns | 4.62 ± 0.55^a,b^ | * | 0.99 ± 0.07^b,c,d^ | * | 0.78 ± 0.07^b,c^ | ns |
|  |  | s | 0.07 ± 0.00^u,v^ |  | 11.72 ± 1.21^y^ |  | 0.84 ± 0.12^x,y^ |  | 0.77 ± 0.04^x,y^ |  |
| ***Lb*. *delbrueckii*** | **UCLM-32** | p | 0.07 ± 0.00^a^ | ns | 4.70 ± 0.54^a,b^ | ns | 1.10 ± 0.08^b^ | ns | 0.74 ± 0.01^a,b,c^ | * |
|  |  | s | 0.07 ± 0.01^t,u^ |  | 4.33 ± 0.15^t^ |  | 1.08 ± 0.05^u^ |  | 0.68 ± 0.02^t,u^ |  |
| ***L*. *brevis*** | **UCLM-99** | p | 0.06 ± 0.00^a^ | ns | 4.61 ± 0.05^a,b^ | * | 1.06 ± 0.08^b,c^ | * | 0.78 ± 0.01^b,c^ | * |
|  |  | s | 0.06 ± 0.00^v,w^ |  | 4.94 ± 0.00^t,u,v^ |  | 1.23 ± 0.00^t^ |  | 0.80 ± 0.00^y,z^ |  |
|  | **UCLM-111** | p | 0.05 ± 0.01^b^ | ns | 5.91 ± 0.79^c,d^ | ns | 0.76 ± 0.03^e,f^ | ns | 0.82 ± 0.04^b,c^ | * |
|  |  | s | 0.05 ± 0.00^w,x^ |  | 5.49 ± 0.05^u,v,w^ |  | 0.77 ± 0.01^y^ |  | 0.66 ± 0.01^t^ |  |
| ***Lp*. *plantarum*^#^** | **299v** | p | 0.05 ± 0.00^b^ | * | 5.62 ± 0.47^b,c^ | * | 0.80 ± 0.20^d,e,f^ | * | 0.73 ± 0.12^a,b,c^ | ns |
|  |  | s | 0.07 ± 0.00^t,u^ |  | 4.40 ± 0.04^t^ |  | 1.23 ± 0.02^t^ |  | 0.78 ± 0.02^x,y^ |  |
|  | **CECT 7315** | p | 0.07 ± 0.01^a^ | ns | 4.39 ± 0.56^a^ | ns | 1.29 ± 0.08^a^ | ns | 0.77 ± 0.07^b,c^ | ns |
|  |  | s | 0.07 ± 0.00^t^ |  | 4.08 ± 0.22^t^ |  | 1.17 ± 0.19^t^ |  | 0.78 ± 0.02^x,y^ |  |

^#^ *Lp. plantarum* 299v (Protransitus Lp®) and *Lp. plantarum* CECT 7315 (Lactoflora® Protector Inmunitario)

(-) strains were not able to grow

^a-f^ different letters mean significant statistical differences (*p* < 0.05) between strains for the pure form of prebiotic

^t-z^ different letters mean significant statistical differences (*p* < 0.05) between strains for the prebiotic in its commercial supplement form

* significant statistical differences (*p* < 0.05) between the pure form of prebiotic and the commercial supplement form for each strain

ns not significant differences (*p* < 0.05) between the pure form of prebiotic and the commercial supplement form for each strain
